# Radiative pattern of intralayer and interlayer excitons in two-dimensional WS_2_/WSe_2_ heterostructure

**DOI:** 10.1038/s41598-022-10851-3

**Published:** 2022-04-28

**Authors:** Mohammed Adel Aly, Manan Shah, Lorenz Maximilian Schneider, Kyungnam Kang, Martin Koch, Eui-Hyeok Yang, Arash Rahimi-Iman

**Affiliations:** 1 Faculty of Physics and Materials Sciences Center, Philipps-Universität Marburg, 35032 Marburg, Germany; 2 Physics Department, Faculty of Science, Ain Shams University, Cairo, 11566 Egypt; 3 Department of Mechanical Engineering, Stevens Institute of Technology, Hoboken, NJ 07030 USA; 4 I. Physikalisches Institut, Justus-Liebig-Universität Gießen, 35390 Gießen, Germany

**Keywords:** Two-dimensional materials, Two-dimensional materials, Two-dimensional materials

## Abstract

Two-dimensional (2D) heterostructures (HS) formed by transition-metal dichalcogenide (TMDC) monolayers offer a unique platform for the study of intralayer and interlayer excitons as well as moiré-pattern-induced features. Particularly, the dipolar charge-transfer exciton comprising an electron and a hole, which are confined to separate layers of 2D semiconductors and Coulomb-bound across the heterojunction interface, has drawn considerable attention in the research community. On the one hand, it bears significance for optoelectronic devices, e.g. in terms of charge carrier extraction from photovoltaic devices. On the other hand, its spatially indirect nature and correspondingly high longevity among excitons as well as its out-of-plane dipole orientation render it attractive for excitonic Bose–Einstein condensation studies, which address collective coherence effects, and for photonic integration schemes with TMDCs. Here, we demonstrate the interlayer excitons’ out-of-plane dipole orientation through angle-resolved spectroscopy of the HS photoluminescence at cryogenic temperatures, employing a tungsten-based TMDC HS. Within the measurable light cone, the directly-obtained radiation profile of this species clearly resembles that of an in-plane emitter which deviates from that of the intralayer bright excitons as well as the other excitonic HS features recently attributed to artificial superlattices formed by moiré patterns.

## Introduction

During the last decade, atomically thin two-dimensional (2D) materials based on transition metal dichalcogenides (TMDC) have attracted immense interest due to their extraordinary light–exciton interaction^1^. Owing to their wide tunability of the band gap across the visible spectral range^2^ and virtually lattice-matching free incorporation into existing device technology platforms^3^, they gained popularity for nanoscale optoelectronics^4,5^, as well as quantum technologies and photonic integration schemes^6–9^, to name but a few examples. The large electronic energy gaps define the optical properties of 2D TMDC monolayers (MLs), which are governed by neutral and charged excitonic species with extraordinarily large binding energy for (Wannier–Mott type) crystal excitons due to their strong Coulomb interaction, reduced dielectric screening and quantum confinement^10–12^. Furthermore, additional excitonic states beyond charged excitons (trions)^13^, such as the biexcitons^14^ as well as other complex states^15,16^, also affect the dynamics and spectral features^17–20^ of the monolayers, particularly for high-quality samples and predominantly at cryogenic temperatures^21–24^.

Vertical van-der-Waals (vdW) heterostructures (HSs)^3,25^, which can be straight-forwardly assembled from TMDC, hBN and graphene, offer exciting opportunities to study exciton physics, as well as novel and extraordinary phases of correlated matter^26^. These features are understood to play a crucial role in developing next-generation (integrated) photonic and (nanoscale) optoelectronic devices with the help of such artificially stacked multilayer-configured ML/few-layer systems. With the observation of superconductivity in bilayer graphene^27–29^ and other exotic quantum states^30–33^ including bands with topological properties^34^, the interlayer twist angle even gave the whole field a new twist, as different investigations have shown recently^35–37^. HSs comprising ML TMDCs with type-II band alignment are particularly attractive for the effective formation of charge-transfer excitons (interlayer excitons)^38–40^ and consecutive efficient dissociation (charge separation towards electronic contacts) in the heterobilayer (HBL) interface system, benefiting from band hybridizations and electronic band edge offsets. These properties are essential in the context of ultrafast photodetection with HS devices and an efficient photovoltaic effect. Moreover, interlayer excitons are strongly bound (*E*_bind._ > 100 meV) and typically persevere at elevated temperatures up to room temperature and exhibit considerably longer lifetimes (in the nanosecond scale, see for instance Refs.^38,39^ than their intralayer counterparts (e.g., neutral ML excitons). Furthermore, they offer the possibility of forming Bose–Einstein-like condensate coherent states^26,41^ through spontaneous coherence formation below a critical temperature for these dipolar out-of-plane excitons, the demonstration of which requires elaborate investigations of temporal and spatial coherence properties of the quantum-degenerate bosonic state.

In the type-II HBLs established by arbitrarily stacked TMDC monolayers (MLs), the interlayer exciton can be formed between electrons and holes present in two different MLs, which come with a layer-to-layer twist-angle degree of freedom that affects their energetics and dynamics^42^. Remarkably, this interlayer state had been well identified both in photoluminescence (PL) and reflection contrast (RC)^43–45^. Furthermore, due to lattice mismatch and twist angles, artificial periodic potentials are induced with a direct impact on the excitonic phase in the TMDC HBLs—by the so-called moiré superlattice^32,46,47^. This moiré-pattern induced periodic potential modulation, which is tunable in terms of supercell length scale by the twist angle, provides a powerful tool, for instance, in configuring quantum phenomena in 2D HSs^27^, such as by tuning the HBL’s electronic structure in and out of flat band situations or tailoring lateral trapping potentials and interaction strengths.

The aforementioned spatial separation between Coulomb-bound electrons and holes (i.e., the spatially indirect nature of excitons) is understood to result in a considerable out-of-plane dipole contribution which differs from the in-plane dipoles within the ML (i.e., the intralayer excitons). Recently, far-field studies were carried out on different TMDC MLs^20,48^, on a single-crystal layered perovskite^49^ and on a 3D crystal such as InSe^50^ to investigate their radiative patterns, i.e., the different out-of-plane excitons’ emission profiles. Driven in part by the distinct excitonic properties of interlayer excitons (X_IL_) and also the fact that there are no such experiments, which focus on clearly disentangling the radiation patterns for different interlayer and intralayer excitons in TMDC heterosystems, a sound understanding and verification of the emission profile for interlayer excitons is sought.

In this study, we provide a direct measurement of the excitonic luminescence for different states (with in-plane and out-of-plane dipole orientations) of our model-type Tungsten-based HBL system, which can be addressed by our angle-resolved photoluminescence technique (ARPL). Moreover, our analysis supported by numerical modeling shows that the PL signatures for WS_2_ and WSe_2_ MLs indeed originate exclusively from in-plane dipoles. In contrast, we unravel with our experiment the out-of-plane nature of charge-transfer exciton signatures obtained by PL at 10 K, which is in agreement with predictions and our modeling data for these interlayer quasi-particles formed across the HBL interface in the WS_2_/WSe_2_ ML–ML HS. Here, clear evidence is given that this radiative pattern of HBL charge-transfer excitons is distinct from that of the previously-found moiré-induced states in this system, the emission profile of which resembles the characteristics of intralayer (ML) excitons, as well as from that of emission from localized states in WSe_2_. This provides an unrivalled means of differentiation between these species present in HBLs.

## Experiment

**Figure 1:**
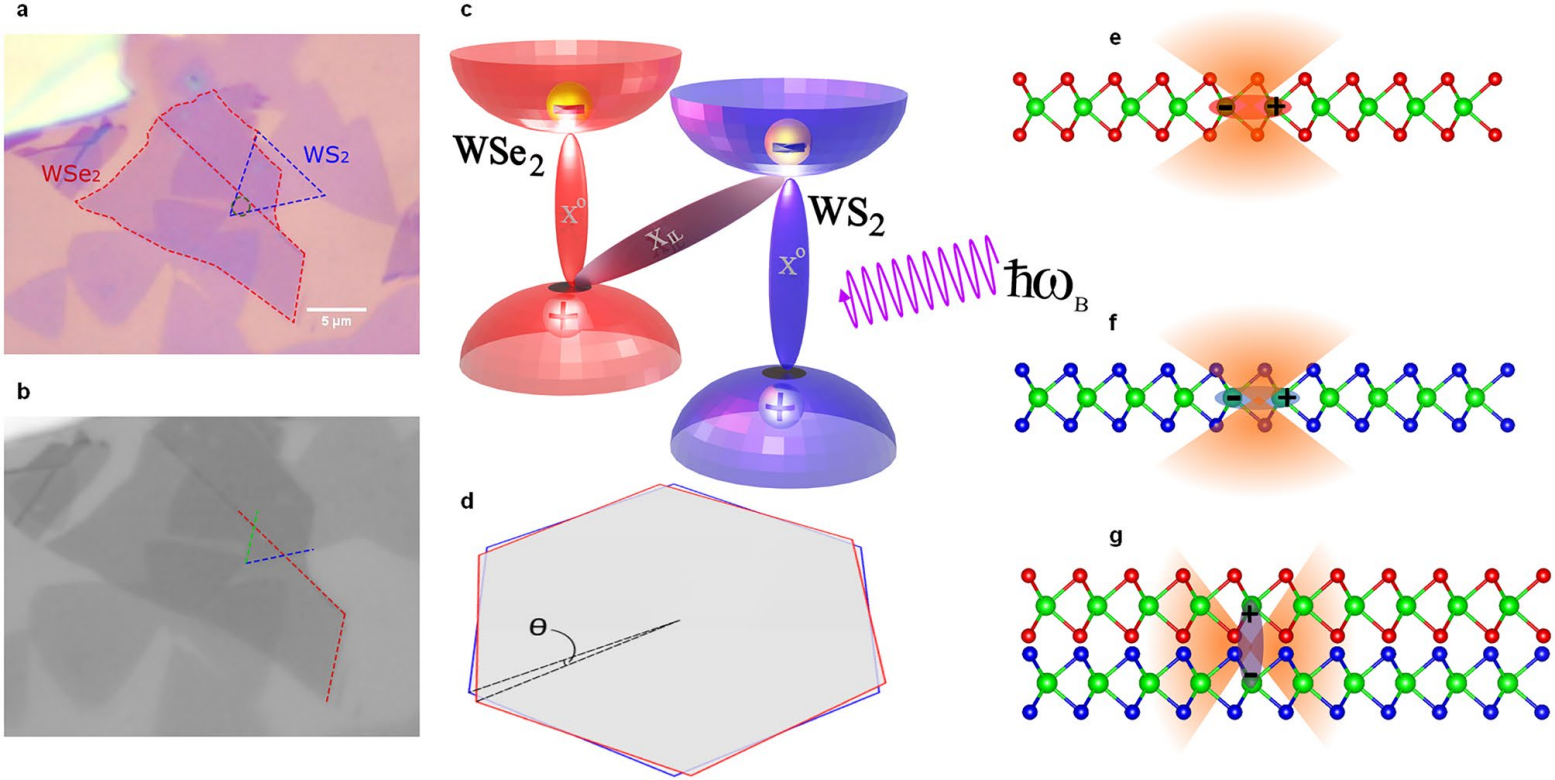
Representation of different excitonic features (transitions) and their corresponding radiative emission patterns of WS_2_/WSe_2_ vdW heterostructure (HS). (**a**, **b**) Optical micrograph for WS_2_/WSe_2_ heterobilayer (HBL) on SiO_2_/Si_2_ substrate (300 nm thermal oxide layer) taken under 100× objective. (**a**) White light color readout. The relevant monolayer (ML) area for WSe_2_ is indicated by the red-dotted frame lines, for WS_2_ by the blue-dotted ones. The dotted circle represents the HBL spot under investigation. (**b**) Intensity profile of the green channel. The red-line marked edges of the top flake and blue/green-lined edges of the CVD (bottom) flake of the HBL region indicate the twist angle θ, here estimated visually to be 56°. (**c**) Schematic drawing of type-II band alignment of the HS. The A-exciton (X^0^) transitions are sketched in red and blue colors for WSe_2_ and WS_2_, respectively. Furthermore, the charge-transfer interlayer exciton (ILX, X_IL_) is indicated as a gray colored transition. (**d**) A sketch of two twisted lattice Brillouin zones in *k*-space is shown, hinting at the possible phase-space mismatch at the corners (representing K and K’ valleys of TMDCs) with increasing angle of θ. (**e**–**g**) In WS_2_ and WSe_2_ MLs, an in-plane dipole orientation for intralayer excitons and their respective out-of-plane radiative emission patterns can be seen. For interlayer excitons, which are formed across the interface of two different HS layers, an out-of-plane dipole orientation and the in-plane radiation pattern is indicated.

In this work, a WS_2_/WSe_2_ HS is studied. The WS_2_ ML was grown by the CVD technique, whereas the WSe_2_ ML was exfoliated from bulk crystal, then stacked over WS_2_ by the dry-stamping technique in order to assemble a HS with a hybrid-production-type HBL region. In Fig. 1, an optical micrograph of the home-built HS is shown as a full-color CMOS-camera image (a) and the gray-scale image of the green channel (b). Red and blue channel micrographs are available in the “Supporting Information” (Fig. SI.2). ML regions and the spot of interest are indicated by dotted lines and labels in the color micrograph, whereas the twist angle can be extracted from crystal flake edge orientations marked in the gray-scale micrograph. Here, the visually-extracted angle amounts to 56° between the lattices. HS and ML characterization by PL, reflection-contrast and Raman spectroscopy is briefly summarized in the “Supporting Information” (Figs. SI.4–SI.7).

As previously reported^43^, WS_2_/WSe_2_ vdW HSs deliver a type-II band alignment between the constituting MLs. Therefore, the electrons from the WSe_2_ conduction band (CB) can efficiently be injected into the WS_2_ CB, provided that the lattice-twist induced phase mismatch at the respective valleys across the interface is not too significant to impede this process—not specifically looking at the impact on the moiré patterns and their implications on the electronic hybridization at this point.

Accordingly, the system ends up with spatially-indirect ILX across the HBL interface with the constituting electron and hole residing in different layers. Thus, the HS exhibits a lower effective optical band gap indicated with the transition for ILX in Fig. 1c. Furthermore, the expected radiation pattern for different intralayer excitonic species (A-exciton in WS_2_ and WSe_2_) and the ILX of this HBL are sketched in Fig. 1e–g. The out-of-plane emission pattern arising from the in-plane dipoles (A-excitons) and the corresponding in-plane pattern associated with the out-of-plane dipoles (ILX) are visualized schematically.

ARPL measurements were performed at a sample temperature of about 10 Kelvin under nonresonant 2.3-eV continuous-wave (CW) laser excitation (532-nm frequency-doubled Nd:YAG) with a spot size of 2–3 µm to examine the radiative nature of different dipole species. The sketched experimental setup^51^ is presented in the “Supporting Information” (Fig. SI.1). In the following, our findings from ARPL for HBL features are examined and summarized.

## Results and discussion

**Figure 2:**
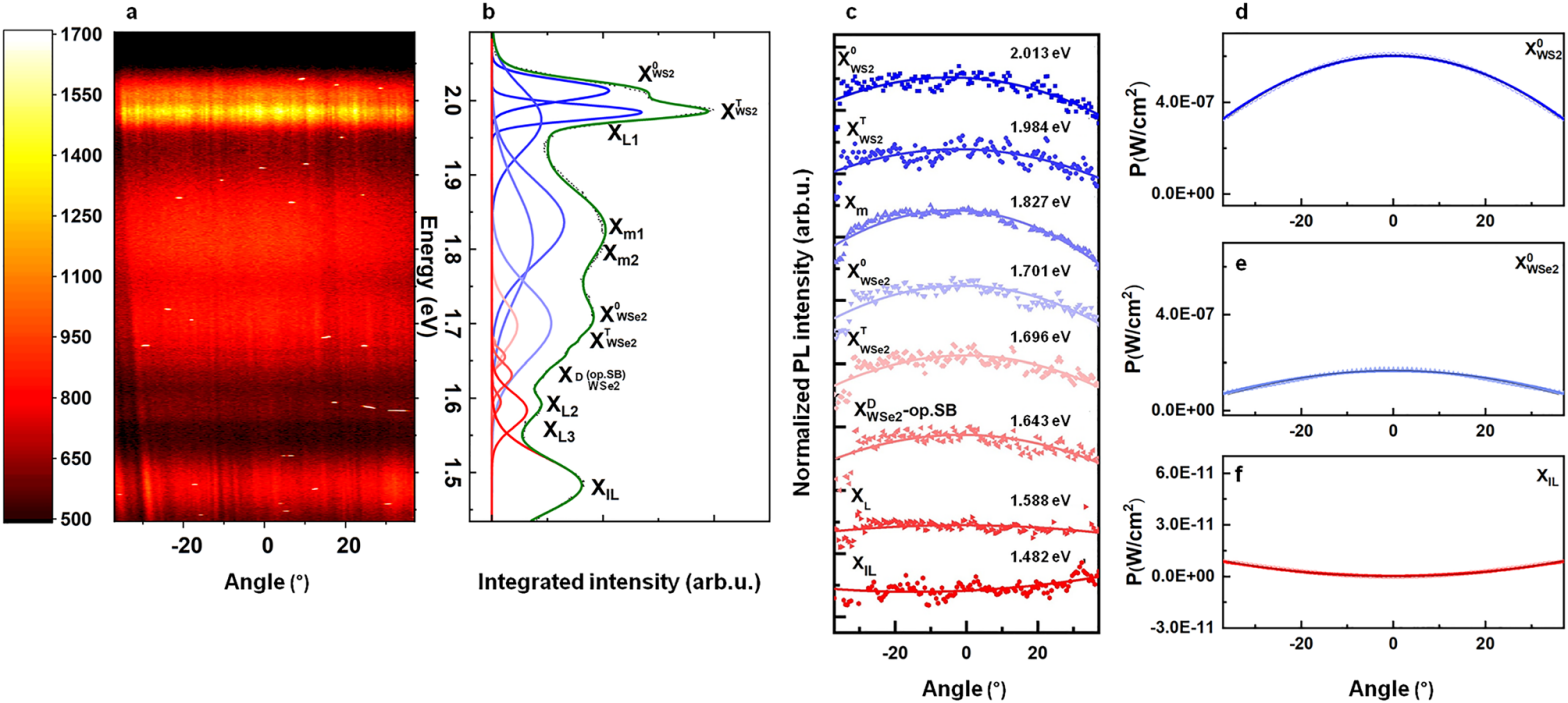
Angle-resolved micro-photoluminescence (ARPL) and their emission pattern. (**a**) ARPL emission spectrum in 2D contour-plot style (linear intensity map) for the WS_2_/WSe_2_ HS at 10 K under nonresonant CW laser excitation. The corresponding angle-integrated line spectrum is depicted to the right (**b**). Recorded data (points) are shown with multi-Gaussian-fit model curves (solid lines, color-coded from blue to red with decreasing mode energy). (**c**) Extracted radiation emission patterns (color-coded data points) for different excitonic species seen in (**a**) and their intensity variation with emission angle. For clarity, a vertical offset is applied. Curved lines are guides to the eyes. (**d**–**f**) Simulated far-field emission (data points) at three different spectral positions, given in P (W/m^2^), for hypothetical in- and out-of-plane dipole emitters (X^0^_WS2/WSe2_ at 2.013 eV/1.70 eV, and X_IL_ at 1.482 eV, respectively) in the simulated HS environment. Solid lines in (**c**–**f**) indicate the differently-strong parabolic trends towards higher angles, as guides to the eyes.

The spectrally-resolved back-focal-plane (*far-field*: FF) PL emission^23,24^ of the different excitonic species is displayed in the contour-diagram intensity spectrum of Fig. 2a. The linear profile scales from dark (low) to white (high counts). In order to extract and analyze different excitonic features, the ARPL spectrum was integrated over the full angle range. The corresponding line-spectrum is plotted in Fig. 2b next to the angle-resolving 2D contour plot in (a). The visible peaks in the summarizing angle-integrated PL intensity spectrum are labeled and represented by fit curves from a multi-peak Gaussian model (Fig. 2b). For clarity, Table SI.1 lists the parameters obtained from Gaussian fitting analysis applied on this line-spectrum. The introduced color code is reused throughout the figure, with blueish and reddish colors representing the higher- and lower-energy features, respectively. According to the inherited convention from previous works, the different excitonic species are labeled at their corresponding energies^16,20,43,52^ (cf. Supporting Information Table SI.1). The neutral/charged intralayer excitons in the respective ML are denoted by X^0^_WS2/WSe2_ and X^T^_WS2/WSe2_, respectively. Here, the spectrum indicates that the WSe_2_ signal is quenched in the HBL region’s emission, and that the interlayer signal is enhanced due to the expected charge transfer (sketched in Fig. 1c). The extracted excitonic features and their energetics in our HBL’s PL agree with those reported by Yu et al.^40^ and Shi et al.^53^ for X^0^, X^T^ in WS_2_ and the HBL’s ILX, denoted X_IL_. Whereas, X_0_, X_T_ in the ML WSe_2_ region and the HBL’s moiré-attributed feature X_m_ agree well with the data reported for the fully exfoliation-based stack by Shah et al.^43^ Moreover, dark excitons X^D^_WSe2_-op.SB (optical-phonon sidebands) match those previously reported by Schneider et al.^20^.

From Fig. 2, one can distinguish the interlayer signal from the other excitonic features due to its lower emission energy at 1.482 eV. Furthermore, the difference in emission patterns extracted from Fig. 2a can be compared. Figure 2c provides an overview of different radiation patterns for all clearly spectrally-distinguishable excitonic features. Simulation data augments the analysis and reproduces these profiles as displayed for the representative dipole species with distinct orientations in Fig. 2d–f. This analysis is performed in a similar fashion as for the bright- and dark-exciton study by Schneider et al.^20^.

In Fig. 2c, the displayed radiation profiles are obtained from spectral integration over their relevant particular energy region (see Table SI.1). Guides to the eyes indicate the curvature. Curvature values are extracted via a phenomenological quadratic fitting to the data (see “Supporting Information Section” and Table SI.1), confirming the observed flection. Here, the interlayer exciton and other excitonic features are discrimitated by their concave and convex intensity distribution along the emission-angle axis, respectively. While basically all recorded species feature a convex shape, i.e., the maximum output centered at normal incidence, the ILX profile reveals an inverted behavior with a concave shape, i.e. the minimum being centered. This behavior is directly attributed to an in-plane emission behavior of the interlayer exciton (out-of-plane dipole orientation) and the emission orientation perpendicular to the 2D-material plane of other excitonic features (intralayer dipoles), respectively, e.g., A-exciton and -trion for the two involved MLs. It is apparent from this graph that most of the excitonic features show a similar behavior as neutral excitons. Markedly, on the other hand, the moiré feature emission is most directional and exhibits the most pronounced convex intensity profile. This can be attributed to the collective emission from the artificial quantum-dots/nanoislands array forming a single wavefront with out-of-plane propagation. Such moiré-induced array defines the 2D-periodic in-plane potential modulation and traps moiré excitons within nanosize supercells, delivering a plethora of in-plane emitters acting as arrayed nanoantennae. In contrast to in-plane and out-of-plane oriented species, PL lines from defect states in the TMDCs, such as the X_L_ signal, expectedly exhibit no observable angle dependencies compared to the other distinct HS exciton types (cf. flat X_L_ intensity profile in Fig. 2c), according to the statistical emission distribution from localized emitters.

To support our experimental findings, an electromagnetic simulation was carried out for two differently-oriented emitter types (in-plane and out-of-plane dipoles) to calculate their far-field pattern (see “Methods” section). Simulated FF patterns, i.e. the angle-dependent irradiance (W/cm^2^) for a given dipole in the (dielectric/semiconducting) host environment at predefined emission energy, are displayed in Fig. 2d–f in a linear intensity scale: the radiation profile for the A-exciton of WS_2_ (d) and of WSe_2_ (e) clearly show the expected maximum emission at normal incidence with an inverse-parabola-like decay to the sides; whereas, the interlayer exciton X_IL_ (f) exhibits an opposite behavior for the same angle range and drastically lower outcoupling from the layered HBL host environment. Thereby, it is clearly demonstrated that the simulated radiation profiles are in a good agreement with the experimental results obtained from the angle-resolved measurements.

This experiment as well as analysis confirms that the obtained emission patterns for the interlayer exciton and the other excitonic features arise from intrinsic out-of-plane and in-plane dipole orientation, respectively, of the Coulomb-bound quasiparticles of the HBL region under investigation.

## Conclusion

In summary, we have analyzed the photoluminescence emission pattern for WS_2_/WSe_2_ van der Waals semiconductor heterobilayers by means of angle-resolved spectroscopy. Our ARPL measurements enable direct access to the emission behavior of different excitonic modes with different dipole orientation in 2D TMDC HBLs and constituent MLs. Our findings highlight the markedly in-plane radiation profile for the interlayer excitons formed from spatially-separated electron–hole pairs across the HS interface, which are distinct in their behavior compared to the other excitonic modes. It is demonstrated at cryogenic temperatures that most of the excitonic modes show a convex angle-dependent intensity profile while the interlayer exciton’s signature is a concave profile. Such information facilitates their utilization in different photonics applications, such as light-based technologies including photovoltaics which may rely on optimized light-matter interactions. This study motivates further *k*-space resolved measurements analysis for interlayer excitons in various type-II heterojunction systems. Moreover, further investigations of angle-resolved HS emission involving external bias across the heterojunction and gate tuning effects are envisioned.

## Methods

### Sample fabrication

The tungsten disulfide WS_2_ monolayers were grown via Low-Pressure Chemical Vapor Deposition (LPCVD) onto an oxidized silicon substrate (~ 300 nm-thick SiO_2_ on Si). A 5 nm-thick tungsten trioxide (WO_3_) was deposited onto the oxidized silicon substrate, sandwiched with another oxidized silicon substrate. The sandwiched sample was loaded into the center of a 3″ quartz tube. For incorporating sulfur (S) in the growth of WS_2_, we placed solid S powder in the furnace tube upstream of the growth area. The ambient gas was purged out to the base pressure of 850 mTorr. As the furnace temperature was ramped at 15 °C/min, WO_3_ was reduced via hydrogenation and subsequent sulfurization of WO_3_. The growth temperature was 900 °C. Ar gas was introduced at 150 °C to reduce moisture. At the same time, H_2_ gas was supplied at 650 °C when increasing the furnace temperature and at 700 °C when decreasing the temperature. The best growth was observed at 4.5 Torr deposition pressure under 60 sccm H_2_ flow rate. The reduction and sulfurization reactions require a higher temperature than the S evaporation. By placing S power at different places on the outside of the central heating area of the furnace, vaporization at different times relative to the substrate temperature can be achieved. At the optimized location for our furnace setup, the S powder was fully consumed after about 30 min from the start of vaporization.

WSe_2_ MLs were exfoliated from a commercial bulk crystal (*2D Semiconductors Inc*) by using the common micro-mechanical-exfoliation and dry-stamping technique. The exfoliated flakes were transferred onto polydimethylsiloxane (PDMS). MLs were selected by optical contrast among the other flakes of different contrast and thickness using an optical microscope. Afterwards, the selected ML was transferred on top of a suitable CVD-grown WS_2_ ML region with the viscoelastic stamp briefly heated to 90 °C for flake release. Thereafter, the PDMS was slightly lifted and the released WSe_2_ ML remained attached to the target. The fabricated HS was then annealed at 300 °C for 4 h under vacuum (~ 10^–6^ mbar) to enhance coupling between the layers.

### µ-PL measurement

Back-focal-plane imaging (i.e., Fourier-space imaging) was performed using a home-built 4-*f* µ-PL confocal optical microscope setup sketched in Fig. SI.1. The Fourier-space spectroscopy technique used in previous works for ML signal characterization^20,23,24^ allows us to measure the far-field PL signal, that is the intensity as a function of the emission angle (Fig. SI.3). Experiments were performed using the 532-nm CW laser as an excitation source with an average power of 1 mW (corresponding to about 31 kW cm^−2^) focused on the sample with a 40× (NA 0.6) microscope objective to a Gaussian spot with a diameter of approximately 2 µm. The main excitonic features and relevant signatures of the examined HBL are preserved over a wide irradiance variation range, while no degradation is observed at such excitation powers when remaining below the typical damage threshold for such samples. The maximum detectable angle corresponds to ± 37°. The sample was mounted in a continuous-flow cryostat at a high vacuum (~ 10^–7^ mbar) and was cooled down to 10 K. The PL emission from the sample was collected by the same objective and directed to the detection optics part. For analysis, a 550-nm long-pass filter was placed after the sample to suppress the laser light in the data acquisition part. The PL signal was mapped using a nitrogen-cooled charge-coupled device (CCD) Si camera attached to the imaging monochromator (*Princeton Instruments Acton SP2300*). By employing the full chip array of the CCD, the angle resolution (± 1°) of the FF signal was obtained.

### Dipole emission simulations

By using *CST Microwave Studio* simulation packages, FF emission patterns for different dipole orientations were simulated. The simulation parameters were extracted from the literature. For WSe_2_, the thickness was estimated to be 0.6 nm^20^. In order to simulate and calculate the emission profile, the Lorentz model was employed. The dispersion parameters for the Lorentz model were extracted from Laturia et al.^54^. For WSe_2_, the in-plane permittivity is ϵ_(∞,z)_ = ϵ_(s,z)_ = 7.5. While for the out-of-plane permittivity, the following values were used ϵ_(∞,x,y)_ = 15.1, ϵ_(s,xy)_ = 15.3, together with a damping frequency of 4.77 THz. Furthermore, for WS_2_, the in-plane permittivity is ϵ_(∞,z)_ = ϵ_(s,z)_ = 6.3, whereas for out-of-plane permittivity, the following values were used ϵ_(∞,x,y)_ = 13.6, ϵ_(s,xy)_ = 13.7, together with a damping frequency of 4.77 THz. The contribution of the out-of-plane intrinsic oscillators (out-of-plane intraband excitons) to the permittivity is much weaker than that of the in-plane component, giving no significant contribution to the permittivity^20^. Therefore, this contribution was neglected for the simulation. For silicon and silicon oxide, values were extracted from the program library. The resulting FF patterns were analyzed by plotting cartesian plots of power flow at constant Azimuth without any means of polarization (absolute value).

### Visualization

The schematic depiction of the WS_2_/WSe_2_ HS and their constituent MLs in Fig. 1e–g is based on crystallographic data provided by the *Materials Project*^55,56^ and drawn by the *VESTA 3* software^57^.

## Supplementary Information

Supplementary Information.

## Data Availability

The data that support the findings of this study are available from the corresponding author upon reasonable request.
