# Supplementary material for: Radiative pattern of intralayer and interlayer excitons in two-dimensional WS2/WSe2 heterostructure
Source: Sci Rep. 2022 Apr 28;12:6939. doi: 10.1038/s41598-022-10851-3 (PMC9050751; doi:10.1038/s41598-022-10851-3)
Supplement: Supplementary file 1 — Supplementary Information. [file 41598_2022_10851_MOESM1_ESM.pdf]

# Supporting Information

## Radiative Pattern of Intralayer and Interlayer Excitons in Two-Dimensional $WS_2/WSe_2$ Heterostructure

Mohammed Adel Aly[1,2,+], Manan Shah[1,+], Lorenz Maximilian Schneider[1,+], Kyungnam Kang[3], Martin Koch[1], Eui-Hyeok Yang[3,*], and Arash Rahimi-Iman[1,4,*]

[1]*Faculty of Physics and Materials Sciences Center, Philipps-Universität Marburg, Marburg, 35032, Germany*

[2]*Physics Department, Faculty of Science, Ain Shams University, 11566 Cairo, Egypt*

[3]*Department of Mechanical Engineering, Stevens Institute of Technology, Hoboken, NJ 07030, USA*

[4]*1. Physikalisches Institut, Justus-Liebig-Universität Gießen, Gießen, 35390, Germany*

*+These authors have equally contributed to this work*

**Corresponding authors*

### S1. Schematic diagram of the angle-resolving experimental setup

The excitation laser is shone on the sample in a home-built conventional microscope setup [48]. Light is focused and collected with a 40x (NA 0.6) microscope objective (MO) mounted on a three-directional (3D) translation stage, employed for focussing as well as beam adjustments. Typical circular spot-sizes on the sample amount to approximately 2 µm in Gaussian beam diameter. The emitted/reflected signal is collected through the same MO. The backscattered laser light is blocked in the detection path by a long-pass filter. The setup is sketched in **Fig. SI.1**.

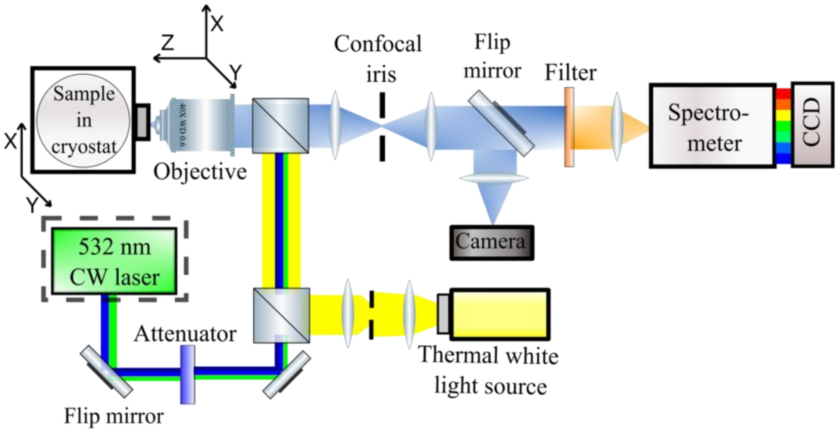


**Figure SI.1 | Schematic diagram of the micro-photoluminescence (PL) and reflection-contrast spectroscopy setup.**

For reflection-contrast measurements, the setup is used with the aid of an iris aperture in the near-field (NF) projection plane (sample's image plane) for spatial selection of the detection area in the order of 1 µm in diameter. The NF signal is further projected onto an imaging spectrometer's entrance slit and detected by an attached liquid-nitrogen cooled Si CCD camera. Moreover, the

sample can be real-time monitored on the high-resolution CMOS (NF) video camera with the help of a flip mirror (**Fig. SI.2**).

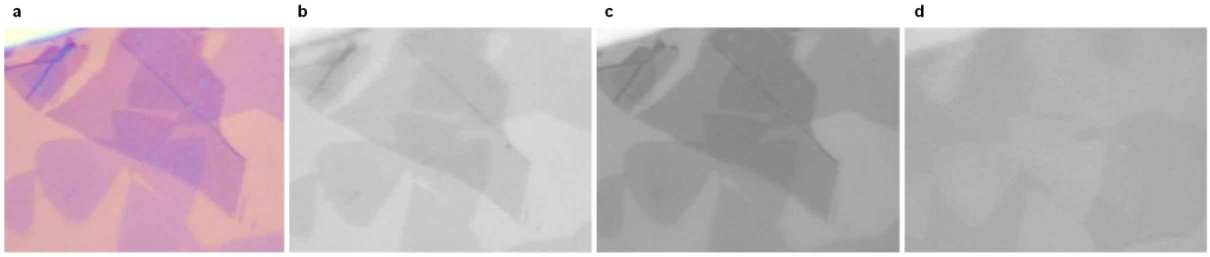


**Figure SI.2 | From left: Optical micrographs in full color and as gray-scale red, green as well as blue channel, respectively.**

For cryogenic measurements, a cryostat and liquid Helium flow can be used to control the temperature between 10 and 300 K. The cooled sample can be laterally translated under the microscope objective with the help of positioning stages.

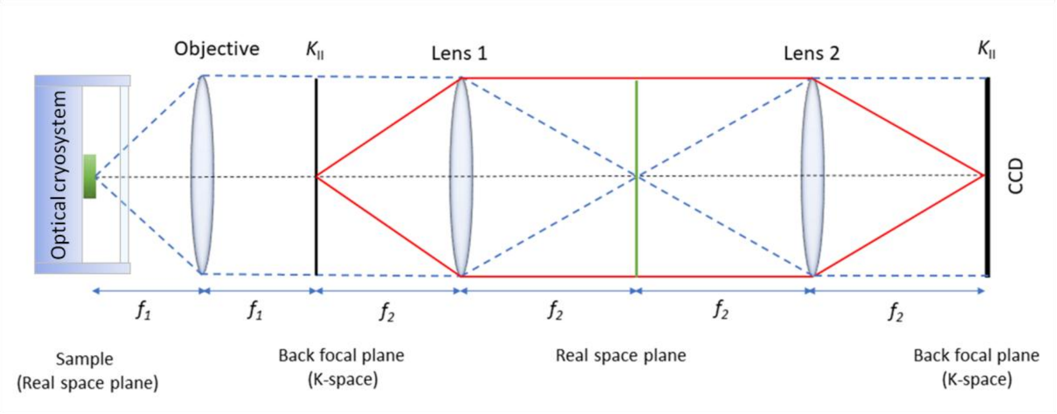


**Figure SI.3 | Sketch of the 4-*f* imaging configuration for angle-resolved spectroscopy.**

To obtain angle-resolved spectra similar to a previous experiment [20], the 4-*f*-projection configuration for Fourier-space imaging (**Fig. SI.3**) is applied which is capable of resolving the momentum space directly onto the imaging-monochromator CCD and previously used for dispersion characterization [23, 24]. The focal lengths of the optics are denoted by the parameter *f*.

## S2. Angle-integrated photoluminescence spectra

In **Fig. SI.4**, two distinct angle-integrated micro-PL (μPL) spectra for the heterobilayer (HBL) are presented, at room temperature (300 K, red) and cryogenic temperature (10 K, black), respectively. The PL spectra are recorded under 2.3-eV continuous-wave (CW) excitation, with imaging spectrometer CCD in line-spectrum mode. The linearly-scaled intensities are normalized and off-set for clarity (intensity in arb. u.). It is clearly seen that the CVD $WS_2$ A-exciton (1.955 eV) (also cf. [S7]) is red shifted compared to the A-exciton for hBN-supported exfoliated samples (~ 2.006 eV) reported before [S1, 40]. This is commonly attributed to strain for CVD samples which results from the growth

of the monolayer (ML) in the furnace at elevated temperature. Furthermore, after growth and during cooling, due to the mismatch of thermal expansion coefficients by one order of magnitude between $WS_2$ monolayer and the $SiO_2$/Si substrate, strain/tension in the ML is imminent [S2]. In short, the $WS_2$ monolayer starts to shrink faster than the $SiO_2$/Si substrate during the cooling process as a consequence, but is stretched due to tension owing to surface adhesion between ML and substrate surface. Such conditions with impact on the band structure and vibrational modes can indeed affect exciton energies in PL spectra and are indicated by Raman mode shifts, respectively. Moreover, the decrease in the bandgap with increasing temperature in comparison to 10-K measurements is explainable through the enhanced electron–phonon interactions at elevated temperatures [S3], accompanied by changes in the bond lengths and evidenced through phonon-side bands as well as polaron formation [50, S4, S5, S6]. Due to the strong coupling between layers after annealing, the multi-exciton features occurring from hybridization of the electronic states of the original monolayer states can be observed and probed experimentally for the HBL region. Moreover, the formation of lower-energy interlayer excitons with their relatively bright emission centered at energies at about 1.48 eV (10 K) is obtained because of the strong overlap between the electron and hole wave functions in the targeted type-II heterosystem comprising ML TMDCs.

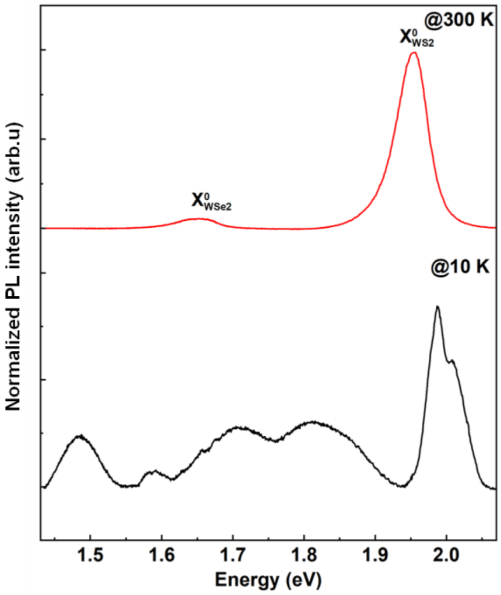


**Figure SI.4 | Angle-integrated µPL spectra for $WS_2$/$WSe_2$ HBL at 300 (red) and 10 K (black).**

## S3. Time-integrated reflection-contrast spectra

To access the properties of the excitonic features over a wide spectroscopic range, white-light reflection-contrast (RC) line-spectra are typically acquired. **Figure SI.5** shows the measured refection contrast (left axis) and its derivative with respect to energy (right axis) for the investigated $WS_2$/$WSe_2$ HBL at (a) 10 K and (b) 300 K. Minimum–maximum normalized RC data are linearly-scaled and offset in intensity. Different excitonic features are labelled for clarity and are in agreement with previous

data for a fully-exfoliation-based HBL [40]. The spectra are obtained after annealing to complement aforementioned µPL data. At 300 K, the A-exciton for both $WS_2$ and $WSe_2$ are clearly visible, and much less pronounced the B-excitons identifiable spectrally in reflection-contrast derivatives. In contrast, at 10 K (**Fig. SI.5a**), considerably sharper lines are recorded, and more subtle resonances are detectable between the excitons for both individual ML materials. While for $WSe_2$ higher-order A-exciton states are indicated above 1.75 eV, its B-exciton mode obscures the clear detection of such states for the $WS_2$ A-exciton above 2.1 eV. Here, the broad ILX resonance below 1.5 eV in RC is washed out in the derivative chart, while signatures from possible moiré features between 1.75 and 1.95 eV may be hidden between the more clearly resolvable A-2s, 3s and higher states of $WSe_2$ in that spectral region. The small feature at 2.1 eV attributed to a B-trion of the $WSe_2$ layer overlaps with the high-energy flank of the $WS_2$ A-exciton, whereas the B-trion of the $WS_2$ layer coincides with the position where the B-trion is expected.

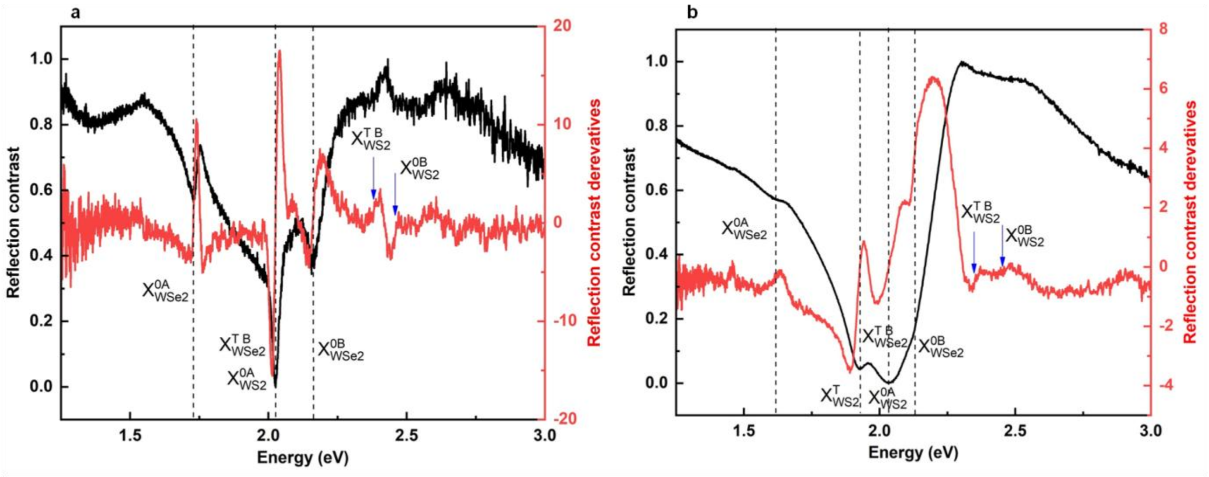


**Figure SI.5 | Time-integrated reflection-contrast data for $WS_2$/$WSe_2$ HBL region at (a) 10 and (b) 300 K**.

## S4. Raman signatures

For the detection of characteristic 2D TMDC Raman modes of the HBL system, a separate commercial Raman setup with a 100x objective (NA 0.9) with 514.8-nm Ar-ion laser as an excitation source is employed. The optical power is kept below 1 mW to avoid sample damage. The signal collected from the same objective is directed through the confocal microscope setup to a spectrometer with 1200-gr/mm grating and a liquid-nitrogen cooled Si CCD [S7]. **Figure SI.6a and b** show Raman modes of the HBL region recorded before and after annealing at 300°C, respectively. Gaussian-curve fits to the experimental data (points) are displayed (solid lines) and the features are labelled for clarity in accordance to the literature [S2, S7].

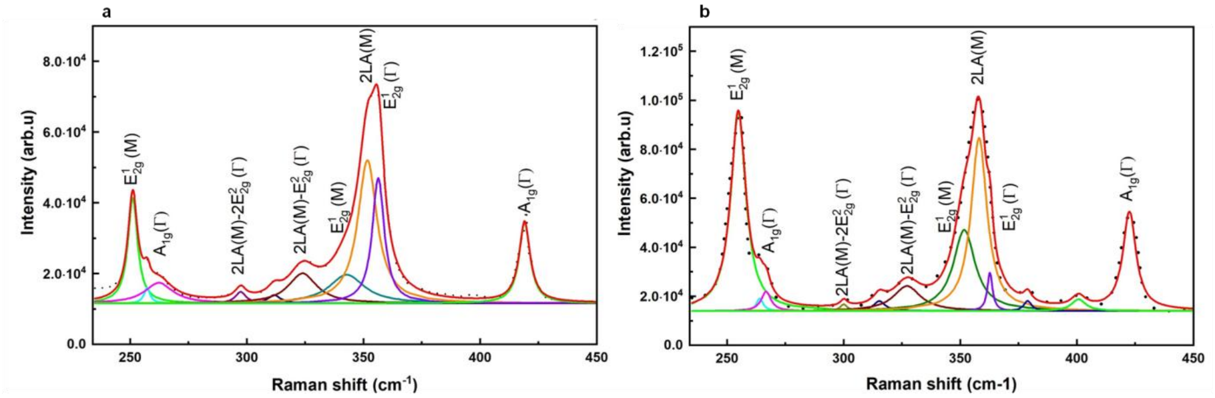


**Figure SI.6 | Raman spectra for the $WS_2/WSe_2$ HBL at room temperature (a) before and after (b) annealing.**

Here, changes in vibrational mode energies due to heterostructure assembly, such as a blue shift detected in the $WS_2$ $E_{2g}{}^1$ mode around 360 $cm^{-1}$ after annealing, are attributed to tension release. Moreover, the blue shift of the $WS_2$ $A_{1g}$ mode towards 450 $cm^{-1}$ might be a result of unintentional doping by adsorption of $O_2$ or $H_2O$, as previously reported [S8, S9]. As the $WS_2$ ML tends to release tension and shrink by adhesion to the deposited $WSe_2$ ML flake, compression strain is transferred to the capping ML of $WSe_2$ as a consequence. Based upon such compression, a blue shift in Raman modes of $WSe_2$ was probed in the literature [S10].

## S5. Excitons' µPL polarization degree for circularly-polarized excitation

To verify the excitonic character of the features in analogy to [S11], measurements with circular-polarized light with excitation–detection configurations $\sigma^+ \rightarrow \sigma^+$ and $\sigma^+ \rightarrow \sigma^-$, respectively, have been performed under CW excitation (at 1 mW). **Figure SI.7** shows PL measurements with different polarization configurations as blue and red spectra, respectively. This sheds light on the circular dichroism over the relevant spectral range. Co- and counter-polarization are established by polarization optics in the excitation as well as detection path in analogy to Ref. [S11]. As expected for excitonic species, which have been previously examined [S11], they exhibit a considerable degree of circular polarization, whereas defect states don't (cf. Refs. [S11, 48]).

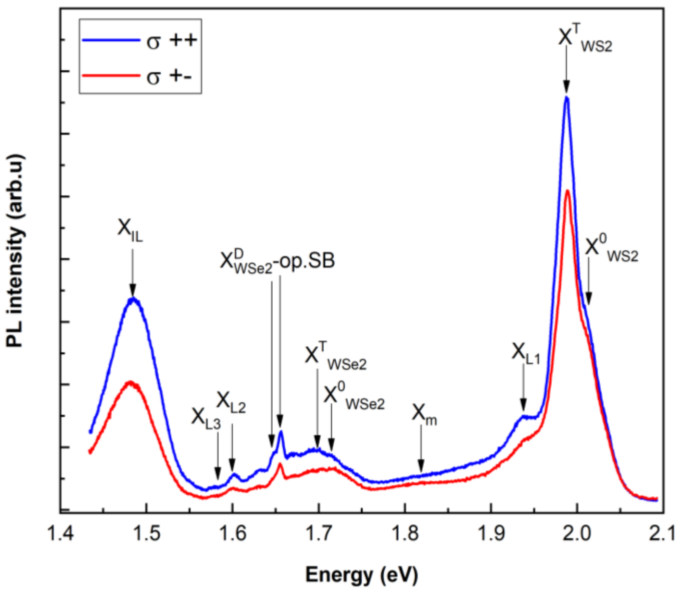


**Figure SI.7 | μPL spectra of co- and cross-polarized signal for the HBL at 10 K. (a), with features labeled as in Table SI.1.**

For clarity and comparability, the different obtainable HBL emission features at 10 K are labeled in the PL spectra according to the convention used in previous reports, at their corresponding energies[20,40,49,50]: The neutral and charged excitons in the individual layers are denoted by $X^0$ and $X^T$, respectively, with a subscript indicating the host material ($WS_2$ or $WSe_2$ here) of the intralayer quasiparticles; $X_{IL}$ and $X_m$ denote the HBL's interlayer and moiré-attributed species, respectively; the spectrally lower-energetic features close to the main exciton modes of an individual ML represent phonon-replica of dark states $X^D_{WSe2}$-op.SB (optical sidebands of $WSe_2$) and localized states $X_L$, respectively. Note that no gray exciton in $WSe_2$ is evidenced in data for the HBL configuration, in contrast to data for hBN-encapsulated ML $WSe_2$ of high quality (see the bright- and dark-exciton study by *Schneider et al.*[20]).

## S6. Curvature analysis for integrated intensities as a function of the emission angle

Curvature analysis has been achieved by selecting a feature's spectral integration width from the spectral data shown in **Fig. 2** and consecutive integration over pixels accommodated in this window in order to obtain an integrated intensity as a function of the emission angle (ARPL profile). Since the certainty of the recorded counts in the spectra is of the order of the scatter between neighbouring pixels and does not affect the extractabilty of curvatures, no additional certainty ranges or error bars are indicated on top of the experimental data points. Followed by a normalization of each feature's ARPL profile to 1 and a vertical offset for clarity, the curved profiles can be displayed as waterfall diagram. To provide a comparison between different ARPL profiles' curvature, a parabolic fit to the individual spectrally-integrated emission profiles (i.e. to the ARPL profiles) has been applied to serve

as “guides to the eyes” in **Fig. 2c**, for the sake of simplicity. The hereby obtainable plain curvature parameter for each species is listed with its fit certainty in **Table SI.1**.

**Table SI.1. Excitonic features of the HBL system extracted from the angle-integrated PL line-spectrum.** Excitonic mode energies and their corresponding linewidths (full-width at half-maximum) are obtained through multi-peak Gaussian-curve fitting. The different excitonic species are labeled as shown in **Fig. 2**. Furthermore, the corresponding spectral integration ranges used to obtain profiles as in **Fig. 2c** are listed. Additionally, the curvature parameter from guides-to-the-eyes parabolic fits to the individual Normalized-PL-Intensity-vs-Angle plots in **Fig. 2c** are summarized together with corresponding fit certainty.

| Excitonic feature | $X^0_{WS2}$ | $X^T_{WS2}$ | $X_{L1}$ | $X_{m1}$ | $X_{m2}$ | $X^0_{WSe2}$ | $X^T_{WSe2}$ | $X^D_{WSe2\ op.SB(i)}$ | $X^D_{WSe2\ op.SB(ii)}$ | $X_{L2}$ | $X_{L3}$ | $X_{IL}$ |
|---|---|---|---|---|---|---|---|---|---|---|---|---|
| Energy (eV) | 2.013 | 1.984 | 1.974 | 1.836 | 1.810 | 1.701 | 1.696 | 1.655 | 1.631 | 1.594 | 1.583 | 1.482 |
| Linewidth (eV) | 0.033 | 0.0244 | 0.100 | 0.130 | 0.167 | 0.118 | 0.070 | 0.023 | 0.035 | 0.026 | 0.055 | 0.087 |
| Spectral Integr. Range (eV) | 2.015 to 1.998 | 1.998 to 1.979 | - | 1.838 to 1.785 | | 1.726 to 1.697 | 1.757 to 1.674 | 1.650 to 1.628 | | 1.601 to 1.568 | | 1.502 to 1.461 |
| Curv. param. ($10^{-4}$, arb. u.) | -3.4 ± 0.3 | -2.9 ± 0.3 | - | -5.1 ± 0.2 | | -4.0 ± 0.2 | -3.6 ± 0.3 | -3.0 ± 0.3 | | -0.8 ± 0.2 | | +0.6 ± 0.2 |

It is worth noting that because the experimental ARPL data also reflects other aspects, such as carrier densities, exciton populations, dynamics and radiative/outcoupling rates, a comparison of signal strength and curvatures between measurements and simulations is not pursued here. Instead, to facilitate comparison between measured ARPL and simulated far-field emission (theoretical profiles of hypothetical in- and out-of-plane dipole emission, supporting this ARPL study), the area A under each model curve in **Fig. 2d-f** was obtained with the help of parabolic fits (with curvature parameter C). These values (related to plots in W/cm², i.e., not normalized intensity) are:

for $WS_2$ A = 0.38, C = -2.0E-6 (C with negligible fit uncertainty); for $WSe_2$ A = 0.10, C = -0.7E-6; and for XIL A = 2.4E-6, C = +0.00006E-6.

In the measured spectrum, the signal strength difference between $WS_2$ and $WSe_2$ excitons lies in the same order as the difference between the respective areas A listed here (for the model irradiances).

In the case of the ILX (**Fig. 2f**), the considerably lower simulated values of angle-dependent irradiance data (for a strictly out-of-plane model dipole) are orders of magnitude lower than the simulated irradiance in the case of the in-plane dipoles (**Fig. 2d,e**). This is understandable given their difference in orientation with regard to the FF detection with maximum collection angle 37° in the simulation.

However, in contrast to this simple dipole modeling, in the experiment, one typically faces altered PL collection efficiencies due to various possible reasons, such as scattering and free-space coupling

rates modified by the surface, substrate, interfaces, and material stacking. The order of magnitude weaker curvature of ILX compared to $WS_2$ or $WSe_2$ excitons in the measurement still reflects the trend that in normal incidence detection with limited collection angle, detection efficiency should be markedly reduced towards 0° (coming from finite angles); in contrast, at very high angles, drastically more signal can be expected (than around normal incidence). We have limited our consideration for clarity to the experimentally accessed angle range.

The fact that the measurable heterostructure signal ILX already exceeds that of individual $WSe_2$ modes and reaches such remarkable signal strength (e.g. well seen in the angle-integrated spectrum of **Fig. 2b**) can be understood as an indicator of the very high number of charge-transfer excitons; or in other words, the ARPL spectrum indicates the abundant and excessive occupancy of ILX states by energetically relaxed charge-carrier pairs, Coulomb-bound across the interface of the HBL structure and with near-to-model-like out-of-plane dipole character.

## S-References

S1. O. Mey, F. Wall, L. M. Schneider, D. Günder, F. Walla, A. Soltani, H. Roskos, N. Yao, P. Qing, W. Fang, and A. Rahimi-Iman, "Enhancement of the Monolayer Tungsten Disulfide Exciton Photoluminescence with a Two-Dimensional Material/Air/Gallium Phosphide In-Plane Microcavity," ACS Nano 13, 5259–5267 (2019).

S2. D. Kumar, B. Singh, P. Kumar, V. Balakrishnan, and P. Kumar, "Thermal expansion coefficient and phonon dynamics in coexisting allotropes of monolayer WS2 probed by Raman scattering," J. Phys. Condens. Matter 31, 505403 (2019).

S3. Y. P. Varshni, "Temperature dependence of the energy gap in semiconductors," Physica 34, 149–154 (1967).

S4. D. Christiansen, M. Selig, G. Berghäuser, R. Schmidt, I. Niehues, R. Schneider, A. Arora, S. M. De Vasconcellos, R. Bratschitsch, E. Malic, and A. Knorr, “Phonon sidebands in monolayer transition metal dichalcogenides,” Phys. Rev. Lett. 119(18), 187402 (2017).

S5. M. Selig, G. Berghäuser, A. Raja, P. Nagler, C. Schüller, T. F. Heinz, T. Korn, A. Chernikov, E. Malic, and A. Knorr, “Excitonic linewidth and coherence lifetime in monolayer transition metal dichalcogenides,” Nat. Commun. 7(1), 13279 (2016).

S6 C. M. Chow, H. Yu, A. M. Jones, J. R. Schaibley, M. Koehler, D. G. Mandrus, R. Merlin, W. Yao, and X. Xu, “Phonon-assisted oscillatory exciton dynamics in monolayer MoSe2,” npj 2D Mater. Appl. 1(1), 33 (2017).

S7. L. M. Schneider, J. Kuhnert, S. Schmitt, W. Heimbrodt, U. Huttner, L. Meckbach, T. Stroucken, S. W. Koch, S. Fu, X. Wang, K. Kang, E. H. Yang, and A. Rahimi-Iman, "Spin-Layer and Spin-

Valley Locking in CVD-Grown AA'- and AB-Stacked Tungsten-Disulfide Bilayers," J. Phys. Chem. C 123, 21813–21821 (2019).

S8. L. Wang, X. Ji, F. Chen, and Q. Zhang, "Temperature-dependent properties of monolayer MoS2 annealed in an Ar diluted S atmosphere: An experimental and first-principles study," J. Mater. Chem. C 5, 11138–11143 (2017).

S9. J. T. Mlack, P. Masih Das, G. Danda, Y.-C. Chou, C. H. Naylor, Z. Lin, N. P. López, T. Zhang, M. Terrones, A. T. C. Johnson, and M. Drndić, "Transfer of monolayer TMD WS2 and Raman study of substrate effects," Sci. Rep. 7, 43037 (2017).

S10. O. Frank, G. Tsoukleri, J. Parthenios, K. Papagelis, I. Riaz, R. Jalil, K. S. Novoselov, and C. Galiotis, "Compression Behavior of Single-Layer Graphenes," ACS Nano 4, 3131–3138 (2010).

S11. Y. You, X. Zhang, T. C. Berkelbach, M. S. Hybertsen, D. R. Reichman, and T. F. Heinz "Observation of biexcitons in monolayer WSe2," Nat. Phys. 11, 477–82 (2015).
